# Softening non-metallic crystals by inhomogeneous elasticity

**DOI:** 10.1038/s41598-017-09453-1

**Published:** 2017-09-14

**Authors:** P. R. Howie, R. P. Thompson, S. Korte-Kerzel, W. J. Clegg

**Affiliations:** 1 Department of Materials Science and Metallurgy, 27 Charles Babbage Rd, Cambridge, CB3 0FS UK; 2 Institut für Metallkunde und Metallphysik, RWTH Aachen University, Kopernikusstraße 14, 52074 Aachen, Germany

## Abstract

High temperature structural materials must be resistant to cracking and oxidation. However, most oxidation resistant materials are brittle and a significant reduction in their yield stress is required if they are to be resistant to cracking. It is shown, using density functional theory, that if a crystal’s unit cell elastically deforms in an inhomogeneous manner, the yield stress is greatly reduced, consistent with observations in layered compounds, such as Ti_3_SiC_2_, Nb_2_Co_7_, W_2_B_5_, Ta_2_C and Ta_4_C_3_. The mechanism by which elastic inhomogeneity reduces the yield stress is explained and the effect demonstrated in a complex metallic alloy, even though the electronegativity differences within the unit cell are less than in the layered compounds. Substantial changes appear possible, suggesting this is a first step in developing a simple way of controlling plastic flow in non-metallic crystals, enabling materials with a greater oxidation resistance and hence a higher temperature capability to be used.

## Introduction

Surprisingly low yield stresses have been observed in some non-metallic compounds, with values as low as ~36 MPa^1^ being reported in some layered compounds such as Ti_3_SiC_2_. Understanding why such easy plastic flow occurs in some non-metallic compounds is a starting point in learning how to control plastic flow in non-metallic and normally brittle materials. If this could be achieved, compounds that are more resistant to oxidation could be used. As oxidation often limits the maximum use temperature of a material, this would enable the development of materials that can operate at higher temperatures than are currently possible. In metallic glasses plastic flow has been associated with a variation in the elastic modulus with position in the crystal, though it was thought that this could not occur in crystals, nor was there any explanation of the effect^2^.

We begin by considering crystalline Ti_3_SiC_2_, which may be thought of as being composed of M–X and M–A layers, where M is Ti, A is Si and X is C. Calculations have shown that in these layered structures there is a tendency for electron density in the M–A bond to be drawn toward the M–X layer^3–5^. This might also be expected simply by considering the relative average electronegativities of the two layers^6^. Other layered structures, Nb_2_Co_7_^7, 8^, W_2_B_5_^9^, Ta_4_C_3_^10^ also show anomalously low yield stresses, albeit on a single slip-plane, suggesting that such an effect might be more general. Calculations by density functional theory predict that a layered electronic structure should form in Ti_0.5_Mo_0.5_N and Ti_0.5_W_0.5_N^11, 12^, the effect increasing as the valence electron concentration increased. Furthermore, calculations of the macroscopic elastic constants suggested an increase in the ease of plastic deformation, inferred from a decrease in the value of *G*/*B*, where *G* is the shear modulus and *B* is the bulk modulus, a criterion for plastic deformation first introduced by Pugh^13^.

### Plastic deformation in crystals

Plastic deformation in crystals generally occurs by the movement of line defects, known as dislocations^14–17^. These can be formed, for instance, by inserting an extra half-plane of atoms into a crystal, an *edge dislocation*, that moves on a given plane and in a given crystal direction. Alternatively a sheared region can be incorporated, so that lattice points are transformed into a helix, a *screw dislocation* ^18^. The incorporation of this half-plane or sheared region gives rise to a misfit energy localized in a small region. In most brittle crystals, and even in some metals, e.g. Fe at low temperatures, it is these changes in the misfit energy, as the dislocation moves, that are the predominant obstacle to dislocation motion^19^. These arise from the atoms in the crystal lattice being displaced from their equilibrium positions, so this effect is known as the *lattice resistance*.

**Figure 1:**
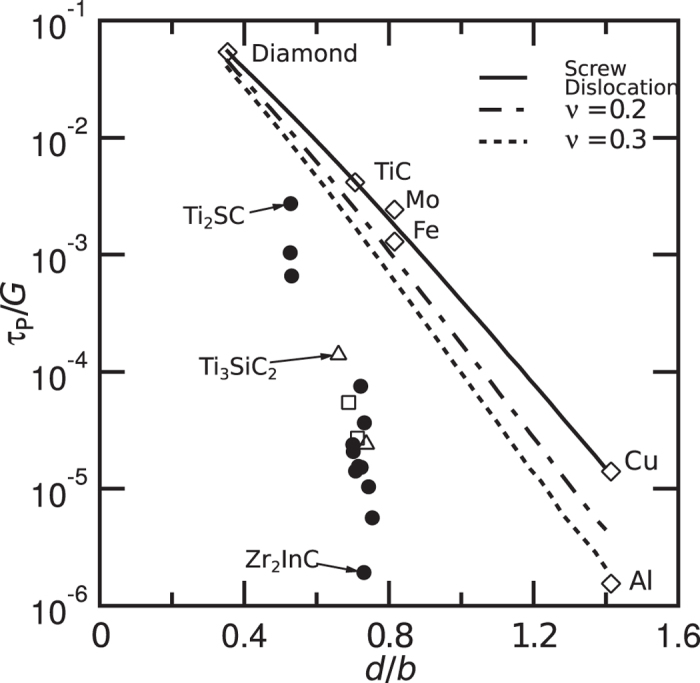
Change in predicted values of τ_P_/*G* with *d*/*b* for some layered compounds compared with experimental values for other materials. The lines show the variation in the predicted values of the lattice resistance at 0 K (Peierls stress), using the approach described in the Methods, for the movement of a screw dislocation and edge dislocations where the material has a Poisson ratio of 0.2 and 0.3 respectively. This is compared with values for some selected materials. Also shown are the predicted values of the Peierls stress, divided by *G*, for different layered compounds. Note that the Peierls stress for the layered compounds decreases with *d*/*b* more rapidly than predicted by the Peierls analysis, suggesting the occurrence of some extra effect. Filled circles denote layered compounds with 211 stoichiometries, e.g. Ti_2_SC, open triangles 312 stoichiometries, e.g. Ti_3_SiC_2_ and open squares 413 stoichiometries, e.g. Ti_4_AlN_3_. The named compounds above show the values of the Peierls stress obtained from the literature.

There are two components to the changes in misfit energy as an edge dislocation moves. Those associated with the misalignments of atoms across the slip plane, *misalignment energies* ^20^: These localize the misfit strains. There are also energies associated with the misfit strains parallel to the slip-plane, compressive in the plane above the slip-plane and tensile below it, *in-plane strain energies*: These act to spread the misfit strains over a larger region. The action of these two terms, one tending to localize the misfit strains, the other tending to spread them, gives rise to an energy minimum, which defines the atom configuration and hence the width of this locally strained region, known as the dislocation width, *w*_0_. This was first quantified by Peierls^21^, who showed that the stress required to move a dislocation in this way, known as the *Peierls stress*, τ_P_, and often given as a fraction of the shear modulus *G*, was dependent on the exponential of the dislocation width, *w*_0_: a wider localised region enabling a dislocation to move more easily. The Peierls stress is also dependent on the atom spacing normal to the crystal plane on which slip occurs, *d*, to that parallel to it, *b* ^21^, with the stress required for dislocation motion through the crystal lattice, in the absence of thermal activation, varying exponentially with the ratio of *d*/*b* ^22^, Fig. 1. The sensitivity of this dependence means that making small changes could have a substantial effect on the magnitude of the lattice resistance.

Previous work on modifying the lattice resistance by doping in halides^23^ and in compound semiconductors^24^ either hardened the material or produced only a relatively small effect. Increasing toughness requires that the stress required for a dislocation to move must be substantially reduced. In this case we wish to decrease the energy associated with the misalignments across the slip plane, which act to reduce the dislocation width, *w*_0_. These energies scale with the shear modulus. Softening requires an increase in the dislocation width and a decrease in these energies, which can be achieved by decreasing, relative to that in adjacent layers.

### The nature of elastic deformation

We therefore start by considering elastic deformation. The shear moduli of the M–X and M–A layers, *G*_M–X_ and *G*_M–A_ respectively, were calculated using density functional theory. The shear modulus of the M–X layer was always greater than that of the M–A layer, suggesting that the unit cell might undergo a non-uniform elastic strain. Such non-uniform strains have been suggested in the tetrahedrally-bonded compound semiconductors^25–27^.

**Figure 2:**
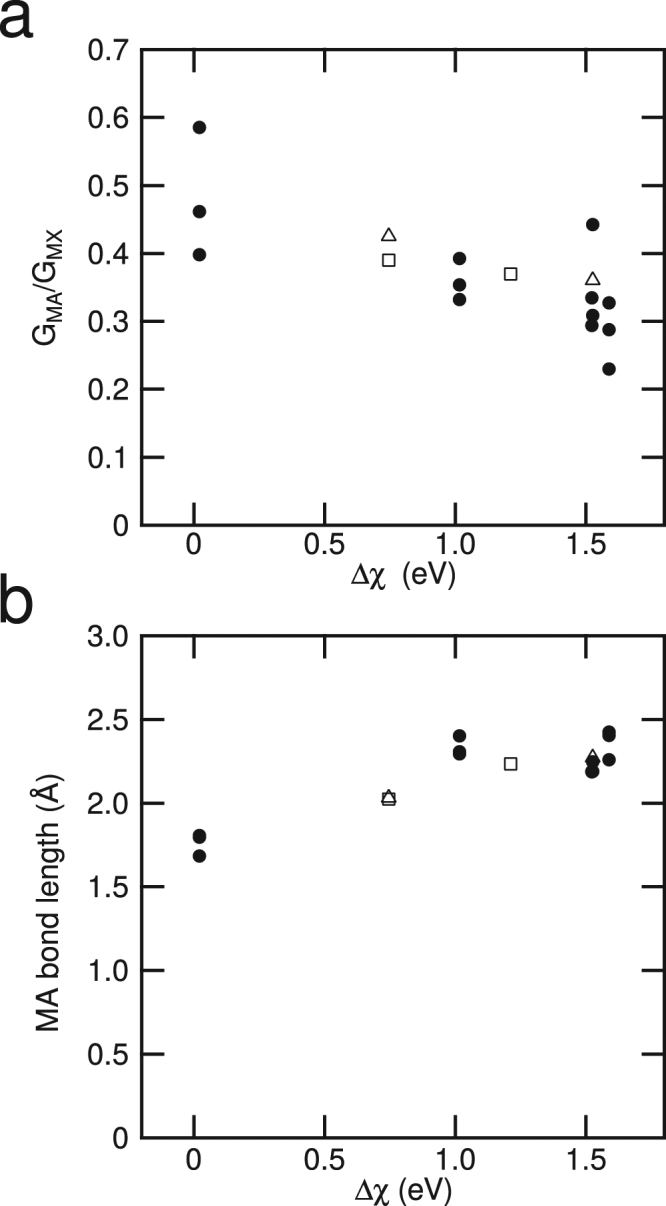
The variation in shear modulus with the electronegativity difference, Δχ, between the M–X and M–A layers. **(a)** Note the increasing value of *G* with increasing Δχ, due to the tendency for electron density to be withdrawn from the M–A layer. **(b)** The variation in the length of the M–A bond with the difference in electronegativity of the M–A and M–X layers. Where electrons are drawn out of the M–A layer, at higher values of Δχ, the M–A bond length also increases.

The electronegativity difference, Δχ, between the layers is equal to

1 $${\rm{\Delta }}\chi ={\chi }_{M{\textstyle \text{-}}X}-{\chi }_{M{\textstyle \text{-}}A}$$

where *χ*_Μ–Χ_ is the arithmetic mean of the electronegativities of M and X, as there are equal numbers of M and X atoms in the M–X layer. Similarly for *χ*_M–A_, as there are also equal numbers of M and A atoms in the M–A layer. The Mulliken scale of electronegativity was used here^28^. If the electronegativity of the M–X layer were greater than that of the M–A layer, electron density would tend to be drawn toward the M–X layer. This occurs where the M–A layers contain elements such as In, Si, Ga or Al. However, if the M–A layer contains S, the electronegativity difference between the layers is much smaller, so that one would expect changes in electron density to be less marked, Fig. 2a.

### The shear modulus of layered structures

These changes in shear modulus were also reflected in changes to the length of the M–A bond, with the bond length increasing as the electronegativity difference between the layers increased, Fig. 2b, and electron density was drawn from the M–A bond.

The difference in elastic constants of the two layers suggests that the overall shear modulus of the unit cell of a crystal might be considered as two slabs: an approach commonly used to describe the elastic properties of elastically inhomogeneous composite materials^29^. Making the assumption that the stresses are the same in two slabs bonded together, the value of the composite shear modulus *G*_slab_ is given by^29^

2 $${G}_{{\rm{s}}{\rm{l}}{\rm{a}}{\rm{b}}}={[\frac{{f}_{{\rm{M}}{\textstyle \text{-}}{\rm{X}}}}{{G}_{{\rm{M}}{\textstyle \text{-}}{\rm{X}}}}+\frac{{f}_{{\rm{M}}{\textstyle \text{-}}{\rm{A}}}}{{G}_{{\rm{M}}{\textstyle \text{-}}{\rm{A}}}}]}^{-1}$$

where *f*_M–X_ and *f*_M–A_ are the volume fractions of the M–X and M–A layers respectively. The values were obtained from the unit cell of each compound^30^. The value of *f*_M–X_ = 4*z*_1_, *z*_1_ being the fractional height of the M_1_ atom in the unit cell and *f*_M–A_ = 1−4*z*_1_ for a compound with a 211 stoichiometry, e.g. Ti_2_AlC, Fig. S1. For 312 and 413 stoichiometries, e.g. Ti_3_SiC_2_ and Ti_4_AlN_3_, *f*_M–A_ = 4*z*_2_ and *f*_M–X_ = 1−4*z*_2_, where *z*_2_ is the fractional height of the M_2_ site in the unit cell, Fig. S1.

**Figure 3:**
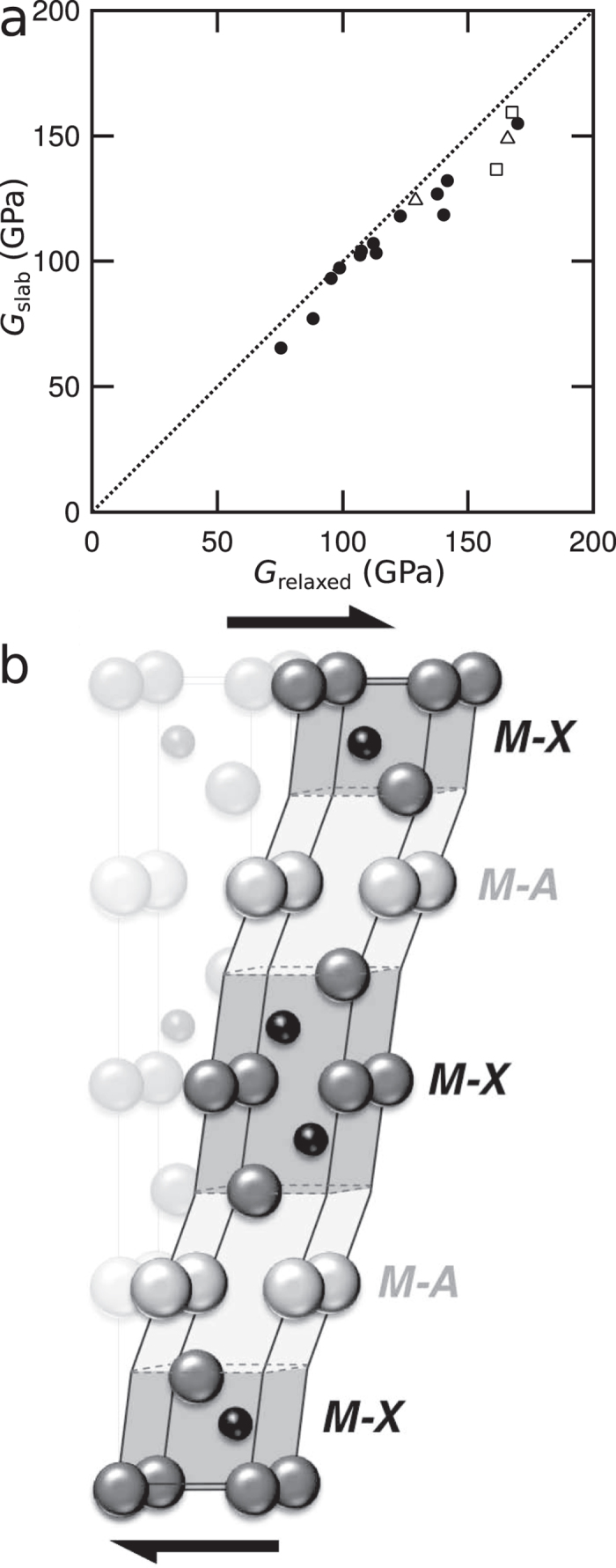
(**a**) Comparing the predictions of a slab model, using the calculated shear moduli and thickness fractions for the individual M–X and M–A layers with the overall relaxed shear moduli predicted. Filled circles denote phases with 211 stoichiometries, e.g. Ti_2_SC, open triangles 312 stoichiometries, e.g. Ti_3_SiC_2_ and open squares 413 stoichiometries, e.g. Ti_4_AlN_3_. The good agreement between the two suggests that such layered compounds elastically deform in a non-uniform way with the more compliant M–A layers deforming more than the stiffer M–X layers, as shown schematically in (**b**).

Despite the simplicity of the approach, the slab model predicts the shear modulus of the layered structure from that of the components surprisingly well, Fig. 3a, giving good agreement between the calculated values using the slab model, obtained from Equation (2), and the calculated values of the relaxed shear modulus. This is also consistent with calculations elsewhere of the bulk modulus^31^. This supports the idea that the unit cell of such a layered crystal elastically deforms in a non-uniform fashion, Fig. 3b, with the more compliant parts of the unit cell deforming more than the stiffer ones, consistent with the apparent scatter in elastic properties that has been observed^5^.

As a result, the calculated value of the relaxed shear modulus is strongly influenced by the modulus of the more compliant M–A layer. This follows from the equal-stress assumption in the slab model^29^. It is the influence of this more compliant layer and the composite nature of the modulus on the atomic scale that has given rise to the difficulties in correlating the overall elastic properties with features such as the electronic structure^5^.

The next question is whether such non-uniform elastic deformation can influence the stress required for the movement of dislocations. The Peierls stress is calculated using the program *Peierls Calculator*, together with values of the variables given in Table S1 in the supplementary material. While thermal activation will modify the exact values measured, it is found that the predicted Peierls stress varies over two orders of magnitude, from (4.07 × 10^–3^) *G* for Ti_2_SC to (1.29 × 10^–5^) *G* for Zr_2_InC, Fig. 1. In terms of absolute stresses, this ranges from ~1 MPa for Zr_2_InC to ~690 MPa for Ti_2_SC. This range in Peierls stress corresponds approximately with that for copper, a tough metal, at the lower end, whereas at the higher end they are similar to those for TiC and TiN, materials used for their high hardness, Fig. 1. A large variation is therefore possible, suggesting that the approach might be a useful one.

## Discussion

**Figure 4:**
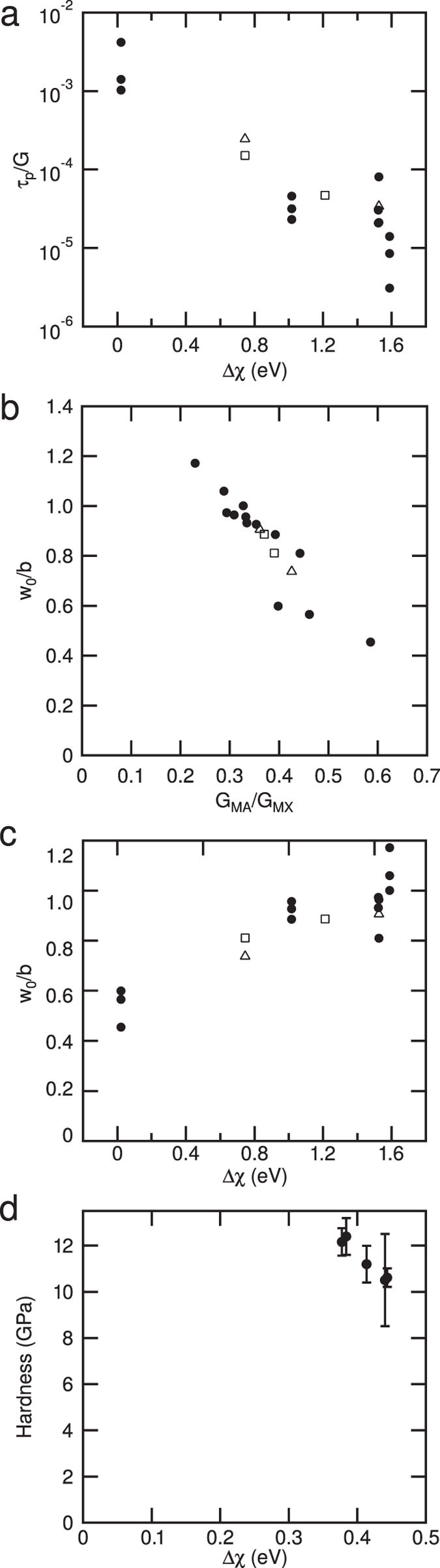
The influence of the electronegativity difference between the M–A and M–X layers on the Peierls stress as a fraction of the shear modulus. (**a**) Note that as the electronegativity difference increases, electron density is drawn from the M–A layer, and the Peierls stress decreases. (**b**) Variation of the dislocation width, *w*_o_/*b*, with the ratio of the shear moduli in the M–A and M–X layers. Note that these are greater than the values obtained in a material that deforms uniformly. (**c**) The change in estimated dislocation width, *w*_o_/*b*, with the electronegativity difference between the the M–A and M–X layers. Note the width increases as Δχ increases and electrons are drawn from from the M–A layer. Filled circles denote phases with 211 stoichiometries, e.g. Ti_2_SC, open triangles 312 stoichiometries, e.g. Ti_3_SiC_2_ and open squares 413 stoichiometries, e.g. Ti_4_AlN_3_. (**d**) Shows the effect of a smaller electronegativity difference, relative to the compounds considered above, on the hardness of a complex metallic alloy with the cubic Ti_2_Ni stucture, with the compositions: Ti_2_Ni, Ti_2_Co, Hf_2_Co, Ti_2_(Co, Ni), (Hf,Ti)_2_Ni.

It is suggested here that the changes in Peierls stress arise due to changes in electron density brought about by differences in electronegativity between the adjacent layers within the unit cell, giving rise both to non-uniform elastic deformation of the unit cell and changes in *d*/*b*. Incorporating these effects into a modified Peierls model to allow for the non-uniform elastic deformation predicts that the rate at which τ_P_/*G* decreases with *d*/*b* is greater than predicted by the conventional analysis, where the elastic deformation within a unit cell is uniform. This indicates the existence of some extra effect. Figure 4a shows that the magnitude of the Peierls stress decreases as the electronegativity difference between the M–X and M–A layers increases. This can be understood in the following way. Where the electronegativity difference between the layers causes a shift of electron density, from the M–A layer to the M–X layer, the shear modulus of the M–A layer decreases with respect to that of the M–X layer. As the misfit energies scale with the shear modulus, this reduces the misalignment energies with respect to the in-plane strain energies. This reduction enables the dislocation width to increase, so that the dislocation moves at a lower applied stress, Fig. 4b. No threshold is apparent, unless it is less than 0.02 (Table S1 Supplementary Information), which is most likely within any error, both in the calculation of the dislocation widths and even the electronegativities. Furthermore, one might expect this withdrawal to be a continuous function of the electronegativity so that a threshold would not be anticipated, consistent with the continuous change observed in Fig. 4b.

Conversely, where the electronegativity difference between the M–X and M–A layers is small, that is where the M–A layers contain S, there is little change in electron density, giving rise to a higher Peierls stress caused by a smaller dislocation width, Fig. 4c. Unfortunately, experimental comparisons are difficult, as there are no low temperature measurements of the yield stress to compare with the predicted values of the Peierls stress. However, the yield stress at room temperature, in large grains, has been determined to be ~36 MPa, not far from the 26 MPa predicted here and well within the error of the Peierls calculation^21^. Some micropillar deformation studies do exist^32^. However, there is a difficulty in that in such materials one might expect size effects to be important^33^. This is consistent with measured strengths of the order of 1 GPa, much greater than the values measured in larger samples^1^.

This approach is also consistent with the observation that Ta_2_C has a lower flow stress^34^ than Ta_4_C_3_. Here double layers of Ta atoms lie between layers of TaC, so that one would expect a similar effect to occur as described above. The idea of electronegativity differences within the unit cell giving rise to a low yield stress in layered compounds can also be extended to other layered structures, such as Nb_2_Co_7_, W_2_B_5_ and Ta_4_C_3_, Supplementary Fig. S2.

### Demonstration in a cubic Ti_2_Ni-type structure

To demonstrate this effect we have measured the hardness in a Ti_2_Ni crystal structure, Supplementary Fig. S3. The cubic Ti_2_Ni structure can be described as ABC stacking of 111 planes, each of which consists of two physically distinct layers, a mixed layer consisting of Ti and Ni sites in the ratio 8:9 and a layer made up entirely of Ti sites. Changing the elements occupying these sites leads to a change in the electronegativity of these two layers. The details are given in the Materials and Methods. A cubic structure is required here so that there are sufficient slip systems that the indent can be accommodated without leading to substantial elastic stresses^35^. However, the range of electronegativity difference, ~0.38–0.44 eV, Fig. 4d, is smaller than in the layered compounds, where the range is 0.02–1.59 eV but even so a decrease in hardness of 2 GPa was observed as electrons are withdrawn from the (111) slip plane.

### Summary

In summary, we have shown that the low yield stresses observed in some crystals are associated with electronegativity differences within their unit cells, giving rise to changes of electron density and hence of local stiffness in different parts of the unit cell. This reduces the magnitude of the energy terms that localize the region of misfit around a dislocation, allowing the width to increase and enabling the dislocation to move more easily. These ideas are consistent with what is observed in other easily deformed crystals. Importantly, they enable a very wide range of behaviour to be obtained. This suggests that using controlled electronegativity differences in crystals offers a first step in a general route to being able to greatly increase the toughness of non-metallic materials.

## Methods and Materials

A number of layered compounds were chosen so that there was a range of electronegativity differences between the M–X and M–A layers, table S1, so that the effects of electron density being drawn toward the M–X layer could be investigated. Compounds selected for study were chosen from those which have already been synthesised experimentally, according to Fig. 4 of Aryal *et al*.^5^. Sixth-row elements were avoided due to difficulties associated with producing pseudopotentials containing occupied *f* states. Where possible, families of compounds were selected so that the effect of changing the M atom, changing the *A* atom or moving from 211 to 312 or 413 structures could be studied. Of particular interest were compounds containing sulphur, which is the sufficiently more electronegative than most A elements that the M–A layer is more electronegative than the M–X layer.

The model used is similar to that described previously^36^, except that the minimum energy configuration is calculated for each step. This causes the changes in misalignment energy to be greater than those associated with the in-plane energies.

To use the Peierls analysis, the generalised stacking fault energy surface, or γ surface is required^37^. This replaces the original assumption made by Frenkel^38^ in calculating the ideal shear strength of a material that the restoring force varies sinusoidally between atoms. In order to insert the γ surface into the Peierls model, it must be represented as a mathematical function. The function chosen is

3 $$\gamma =\sum _{m=1}^{M}{C}_{m}[1-\,\cos (\frac{2m\pi {\rm{\phi }}}{b})]$$

where φ is the displacement and the *C*_*m*_ are coefficients adjusted to fit the DFT results. In most cases, three terms were sufficient. This function can be put into the Program *Peierls Calculator* to estimate the Peierls energy, Peierls stress and dislocation width at the rest position.

This was calculated by displacing two halves of the crystal relative to each other, taking the slip vector as $a/3\langle 11\bar{2}0\rangle$ ^13^. Periodic boundary conditions were used and the simulation cell contained two opposing slip planes. Atoms were allowed to relax in the two directions perpendicular to the slip direction but not in the slip direction itself, otherwise the system would simply return to its equilibrium state. What is being calculated here is the Peierls stress, that is the lattice resistance at 0 K. This will give an upper bound to the yield stress, if it is assumed that other effects are minimal. In this paper, the aim is to see what effects might cause a sudden decrease in the magnitude of the lattice resistance.

The energies associated with misalignments across the slip plane, *misalignment energies*, were determined using DFT, rather than using the Frenkel assumption^38^. These were then fitted to give a variation in the misalignment energy for each atom pair. The slip vector was taken as $a/3\langle 11\bar{2}0\rangle$ ^13^.

Density functional theory (DFT) calculations were carried out using SIESTA, a pseudopotential-based LCAO code^39, 40^. To allow for the possibility of interactions between orbitals of different energies on different elements, semicore pseudopotentials with partial core corrections in the Perdew-Burke-Ernzerhof (PBE) formulation^41, 42^ were generated and tested for transferability using the Troullier-Martins procedure^43^, as implemented in SIESTA’s ATOM code^40^. A double-ζ, polarised basis set, additionally including the semicore states, was used; cutoff radii of the basis functions were optimised using a variational simplex method.

For each compound, initial lattice parameters and atom positions were taken from literature, with experimental values preferred where available^30^. The simulation cell was the conventional *P*6_3_/*mmc* unit cell, with periodic boundary conditions applied in all three directions. The *a* parameter, *c*/*a* ratio and the *z* heights of those layers unconstrained by symmetry were all refined. Where atom positions were relaxed, this was by molecular dynamics, using a conjugate gradient relaxation scheme.

The shear moduli were calculated by calculating the energy of the cell under an imposed strain. This was carried out under three different conditions.

1. With atoms on the symmetrical planes at *z* = 0 and 0.5 fixed but all other atom positions free to relax, giving the relaxed shear modulus, *G*_rel’xd_.
2. With the relative positions of the atoms in the M–X layers fixed, so that the shear distortion is concentrated about the A atoms, giving the shear modulus of the M–X layer, *G*_M–X_.
3. With the relative positions of the A atoms and the first M layers adjacent to them fixed, so that the shear distortion is concentrated in the M–A layers, giving the shear modulus of the M–X layer, *G*_M–A_.

In all cases, the maximum shear strain applied was 2% in the layers, which were free to distort.

Data for the ionization energies and electron affinities are taken from the CRC Handbook of Chemistry and Physics^44, 45^.

### Data availability

The input data used is given in the Supplementary Table S1.

A detailed list is given with the Supplementary Materials and includes the input data used, the programs used to estimate the Peierls stress, structures of various soft crystals and a sample of the input data for the DFT calculations.

## Electronic supplementary material

Softening non-metallic crystals by inhomogeneous elasticity
