# Supplementary material for: Softening non-metallic crystals by inhomogeneous elasticity
Source: Sci Rep. 2017 Sep 14;7:11602. doi: 10.1038/s41598-017-09453-1 (PMC5599516; doi:10.1038/s41598-017-09453-1)
Supplement: Supplementary file 1 — Softening non-metallic crystals by inhomogeneous elasticity [file 41598_2017_9453_MOESM1_ESM.doc]

**Softening non-metallic crystals by inhomogeneous elasticity**

Authors: P.R. Howie^1^, R.P. Thompson^1^, S. Korte-Kerzel^2^, W.J. Clegg^1^* (corresponding)

^1^Department of Materials Science and Metallurgy, 27 Charles Babbage Rd, Cambridge CB3 0FS, UK.

^2^Institut für Metallkunde und Metallphysik, Kopernikusstraße 14, RWTH Aachen University 52074, Germany.

Supplementary Information

1. Supplementary Table S1 showing calculated values.

2. Fig. S1 Crystal structures of the layered 211, 312 and 413 phases. These have chemical formulae respectively of Ti_2_AlC, Ti_3_SiC_2_, and Ti_4_AlN_3_.

3. Fig. S2 Crystal structures of Nb_2_Co_7_, W_2_B_5_ and Ta_4_C_3-_*_x_*.

4. Fig. S3 Crystal structure of Ti_2_Ni type structure

4. Calculation of Peierls stress, including Program Peierls Calculator.

5. Input data for the DFT calculations for Ti_3_SiC_2_.

**Supplementary Table S1.** The electronegativity differences between the layers, Δχ, shear moduli of the M–X, *G*_M–X_, and M–A layers, in GPa, *G*_M–X_ and *G*_M–A_ respectively for some 211, 312 and 413 MAX phases, estimated using DFT. Also given is the modulus, in GPa, assuming uniform deformation, *G*_unif'm_, the relaxed modulus, *G*_rel'xd_, the prediction from the slab model using eqn 1 in the text. The Peierls stress, dislocation width, *w*_o_/*b* and atom spacing, *d*/*b*, and volume fractions of the M–X and M–A layers are also given.

| Phase | Δχ | *G*_unif'm_ | *G*_M–X_ | *G*_M–A_ | f_M–X_ | f_M–A_ | *G*_slab_ | *G*_rel'xd_ | *d*_MA_ | τ_P_/G_rel'xd_ | *w*_o_/*b* | *d*/*b* |
| --- | --- | --- | --- | --- | --- | --- | --- | --- | --- | --- | --- | --- |
| Nb_2_AlC | 1.53 | 163.8 | 230.4 | 101.9 | 0.352 | 0.648 | 126.8 | 137.7 | 2.869 | 6.93 × 10^-5^ | 0.817 | 0.722 |
| Nb_2_GaC | 1.52 | 151.6 | 236.6 | 79.1 | 0.352 | 0.648 | 103.3 | 113.4 | 2.846 | 2.08 × 10^-5^ | 0.932 | 0.702 |
| Nb_2_InC | 1.59 | 152.9 | 251.6 | 82.3 | 0.344 | 0.656 | 107.1 | 112.2 | 2.985 | 1.04 × 10^-5^ | 1.001 | 0.743 |
| Nb_2_SC | 0.02 | 169.9 | 225.1 | 103.9 | 0.396 | 0.604 | 132.1 | 141.8 | 2.586 | 1.04 × 10^-3^ | 0.565 | 0.526 |
| Nb_2_SnC | 1.02 | 142.7 | 229.5 | 81.2 | 0.340 | 0.660 | 104.1 | 107.4 | 2.945 | 2.38 × 10^-5^ | 0.927 | 0.700 |
| Ti_2_AlC | 1.53 | 161.2 | 289.5 | 89.3 | 0.353 | 0.647 | 118.1 | 123.0 | 2.826 | 1.51 × 10^-5^ | 0.967 | 0.723 |
| Ti_2_GaC | 1.52 | 152.6 | 262.2 | 77.0 | 0.352 | 0.648 | 102.5 | 107.0 | 2.788 | 1.42 × 10^-5^ | 0.973 | 0.708 |
| Ti_2_InC | 1.59 | 134.7 | 248.0 | 71.4 | 0.328 | 0.672 | 93.2 | 95.5 | 2.948 | 5.63 × 10^-6^ | 1.060 | 0.754 |
| Ti_2_SC | 0.02 | 179.2 | 220.8 | 129.3 | 0.399 | 0.601 | 154.9 | 169.8 | 2.459 | 3.19 × 10^-3^ | 0.436 | 0.528 |
| Ti_2_SnC | 1.02 | 122.5 | 200.2 | 78.6 | 0.316 | 0.684 | 97.3 | 98.8 | 2.969 | 3.67 × 10^-5^ | 0.886 | 0.732 |
| Zr_2_InC | 1.59 | 125.6 | 209.3 | 48.1 | 0.344 | 0.656 | 65.4 | 75.4 | 3.117 | 1.92 × 10^-6^ | 1.172 | 0.731 |
| Zr_2_SC | 0.02 | 155.3 | 225.4 | 89.7 | 0.405 | 0.595 | 118.6 | 140.3 | 2.641 | 6.60 × 10^-4^ | 0.599 | 0.531 |
| Zr_2_SnC | 1.02 | 122.7 | 179.1 | 59.5 | 0.344 | 0.656 | 77.2 | 88.2 | 3.076 | 1.56 × 10^-5^ | 0.957 | 0.715 |
| Ti_3_AlC_2_ | 1.53 | 164.6 | 230.9 | 83.3 | 0.516 | 0.484 | 124.3 | 129.1 | 2.878 | 2.27 × 10^-5^ | 0.913 | 0.738 |
| Ti_3_SiC_2_ | 0.75 | 192.1 | 241.0 | 102.5 | 0.542 | 0.458 | 148.9 | 165.9 | 2.681 | 1.54 × 10^-4^ | 0.727 | 0.660 |
| Nb_4_AlC_3_ | 1.21 | 209.9 | 262.2 | 95.8 | 0.629 | 0.371 | 159.5 | 167.6 | 2.886 | 2.72 × 10^-5^ | 0.887 | 0.713 |
| Ti_4_SiC_3_ | 0.75 | 198.2 | 215.6 | 84.3 | 0.629 | 0.371 | 136.7 | 161.4 | 2.695 | 5.45 × 10^-5^ | 0.811 | 0.689 |


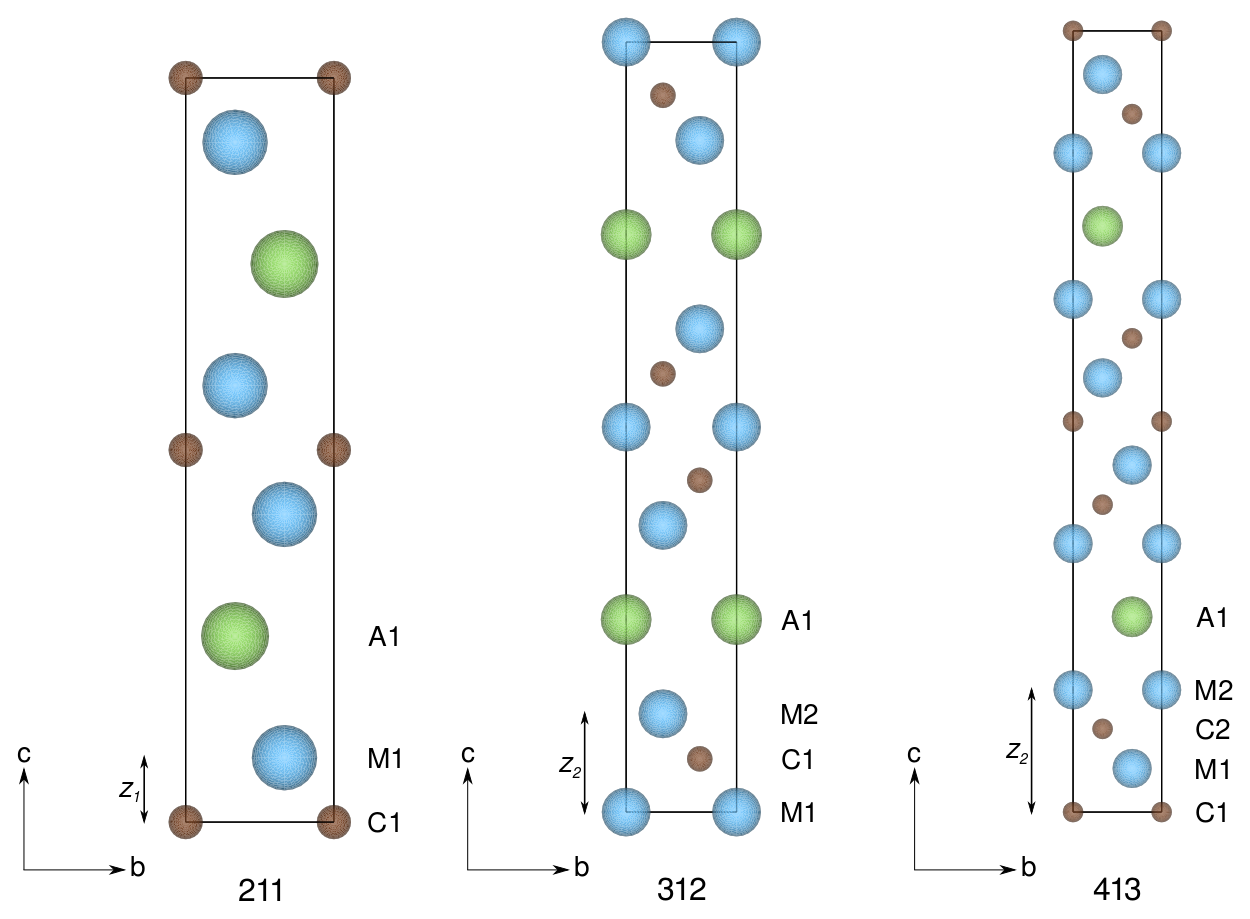
 **Supplementary figure S1**. The three different crystal structures of the layered ternary carbides and nitrides. The stoichiometries are 211, e.g. Ti_2_AlC; 312, e.g. Ti_3_SiC_2_ and 413, e.g. Ti_4_AlN_3_.


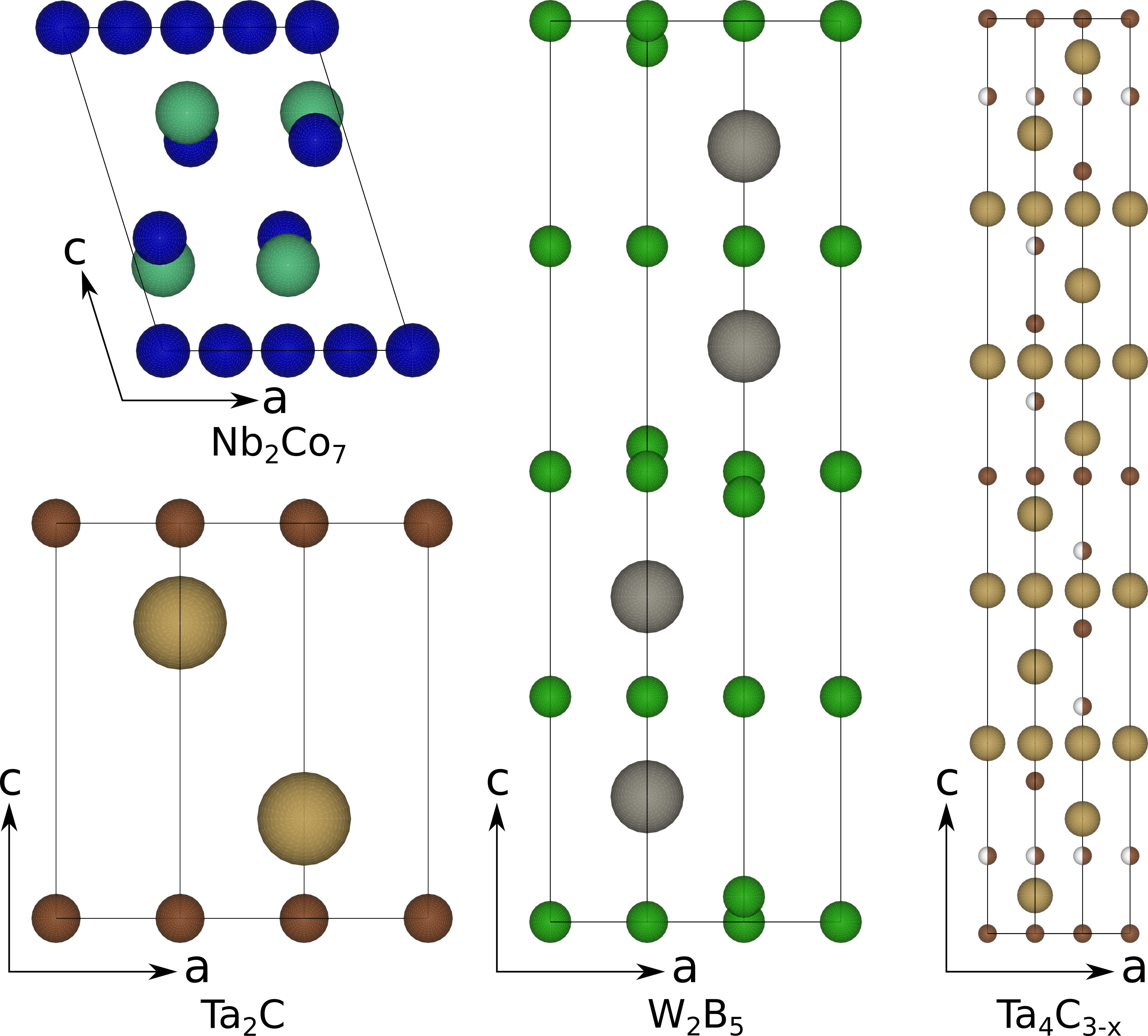


**Supplementary figure S2.** Other crystal structures, Nb_2_Co_7_, W_2_B_5_, Ta_2_C and Ta_4_C_3-_*_x_* that also have a low yield stress on a limited number of directions and crystal planes. The half filled atoms above represent sites with an average occupancy of 50%.


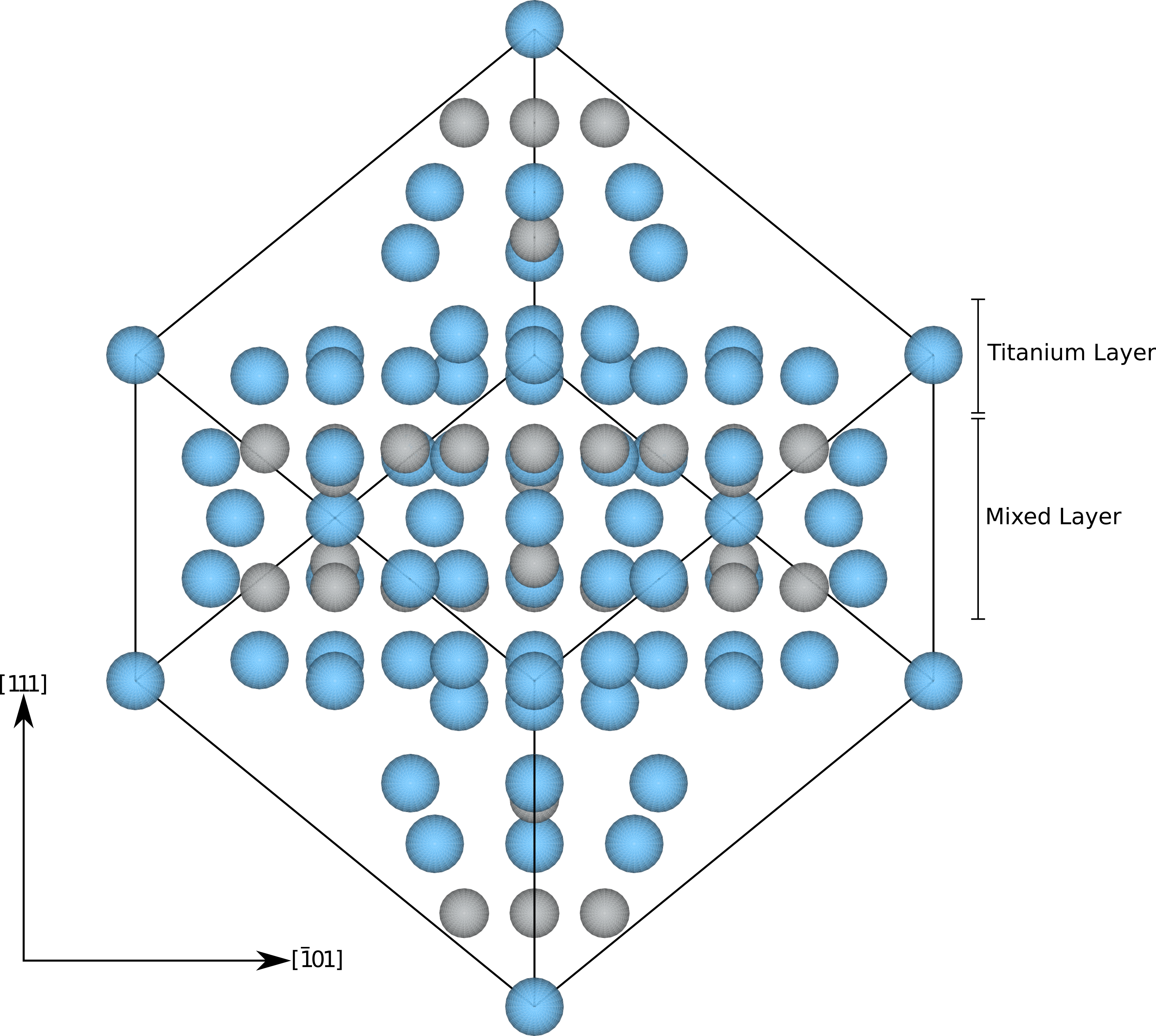


**Supplementary figure S3.** Showing the Ti_2_Ni crystal structure aligned with the slip direction $<\bar{1}01>$ and the slip plane normal <111>, as labelled.

5.' PROGRAM PEIERLS CALCULATOR

' W.J. Clegg, P.R. Howie, R.P Thompson

' A Visual Basic program to calculate the Peierls Stress.

' This program assumes that a spreadsheet is used as the interface to both

' load inputs and display outputs.

Rem Attribute VBA_ModuleType=VBAModule

Option VBASupport 1

Dim amin!

Dim nosteps%

Dim noatoms%

Dim astep!

Dim b!

Dim d_b!

Dim E!

Dim G!

Dim nu!

Dim w#

Dim k#

Dim m#

Dim A1#

Dim A2#

Dim Pi#

Dim a!

Dim xA#()

Dim xB#()

Dim dA#()

Dim dB#()

Dim yA#()

Dim yB#()

Dim Ui0#

Dim Ux0#

Dim Ui#

Dim Ux#

Dim wmin!

Dim wmax!

Dim w1!

Dim w2!

Dim U!

Dim U2!

Dim C1#

Dim C2#

Dim C3#

Dim C4#

Dim C5#

Dim C6#

Sub Calculate()

' Load user inputs, clear the output space

' and redimension arrays to suit the number of atoms

Call LoadInput

Sheet2.Range("A2:G10000").ClearContents

ReDim xA(noatoms)

ReDim xB(noatoms - 1)

ReDim dA(noatoms)

ReDim dB(noatoms - 1)

ReDim yA(noatoms)

ReDim yB(noatoms - 1)

' Put the dislocation in the centre of the crystal

' and calculate the equilibrium energy

a = 0

Call Calcw

Sheet1.Cells(16, 6) = w

Ui0 = Ui * A2

Ux0 = Ux * A1

Call Calcw

Sheet2.Cells(2, 1) = -0.5

Sheet2.Cells(2, 2) = Ui * A2 - Ui0

Sheet2.Cells(2, 3) = Ux * A1 - Ux0

Sheet2.Cells(2, 4) = Ui * A2 + Ux * A1 - Ui0 - Ux0

Sheet2.Cells(2, 6) = w

' Move the dislocation a distance astep, nosteps times

For i = 0 To nosteps

a = amin + i * astep

' Adjust the width of the dislocation to minimise its energy

' This subroutine returns the calculated energies

Call Calcw

' Write the output to Sheet2

' Multiply the energy terms by the pre-factors A1 and A2

' and subtract the equilibrium values calculated above

Sheet2.Cells(i + 2, 1) = i * astep - 0.5

Sheet2.Cells(i + 2, 2) = Ui * A2 - Ui0

Sheet2.Cells(i + 2, 3) = Ux * A1 - Ux0

Sheet2.Cells(i + 2, 4) = Ui * A2 + Ux * A1 - Ui0 - Ux0

Sheet2.Cells(i + 2, 6) = w

Next i

' Calculate the stress by numerical differentiation of the energy

For i = 1 To nosteps

Sheet2.Cells(i + 2, 5) = (Sheet2.Cells(i + 3, 4) - Sheet2.Cells(i + 1, 4)) / (2 * astep)

Next i

End Sub

Private Sub LoadInput()

Pi = 3.14159265358979

nosteps = Sheet1.Cells(2, 6)

noatoms = Sheet1.Cells(3, 6)

amin = -0.5

astep = 1 / nosteps

b = Sheet1.Cells(3, 2)

d_b = Sheet1.Cells(4, 2)

E = Sheet1.Cells(5, 2)

nu = Sheet1.Cells(6, 2)

G = Sheet1.Cells(7, 2)

k = b / (2 * Pi)

' A1 is the pre-factor for misalignment energy

' Includes factor of b/2 to convert between energy per unit area

' and energy per atom line (M5 notes)

' Factor of 10 because energy per unit area in J m^-2, G in GPa, b in Angstroms

A1 = 10 / (2 * G * b)

' A2 is the pre-factor for in-plane strain energy, which multiplies delta^2

' Atom positions already include a factor of b (in Angstroms) so dividing by b^2 is fine

' E and G are both in GPa so no unit correction is required

A2 = E / (2 * G * b ^ 2 * (1 - nu ^ 2)) * d_b

' C1, C2 and C3 are the coefficients of the versed sine terms

' in the expansion for misalignment energy

C1 = Sheet1.Cells(12, 2)

C2 = Sheet1.Cells(13, 2)

C3 = Sheet1.Cells(14, 2)

C4 = Sheet1.Cells(15, 2)

C5 = Sheet1.Cells(16, 2)

C6 = Sheet1.Cells(17, 2)

End Sub

Private Sub CalcEnergy()

x = -Int(noatoms / 2)

' Count along the atom lines

For i = 0 To noatoms - 1

' Atom positions include a factor of b

' xA and xB are the original positions

' a is the dislocation position

xA(i) = b * (x - a)

xB(i) = b * (0.5 + x - a)

' k also includes a factor of b

' dA and dB are the displacements due to the dislocation

dA(i) = -k * Atn(xA(i) / (b * w))

dB(i) = k * Atn(xB(i) / (b * w))

' yA and yB are the final positions

yA(i) = xA(i) + dA(i)

yB(i) = xB(i) + dB(i)

x = x + 1

Next i

'One more atom in A is needed

xA(noatoms) = b * (x - a)

dA(noatoms) = -k * Atn(xA(noatoms) / (b * w))

yA(noatoms) = xA(noatoms) + dA(noatoms)

'Now make another loop to sum the energies

'The atoms in position 0 have no in-plane strain term

'and only one misalignment term between them

Ui = 0

Ux = gamma((yB(0) - yA(0)) / b)

'Most atom positions have an in-plane strain term in each half

'and two misalignment terms

For i = 1 To noatoms - 1

Ui = Ui + (yA(i) - yA(i - 1) - b) ^ 2 + (yB(i) - yB(i - 1) - b) ^ 2

Ux = Ux + gamma((yB(i) - yA(i)) / b) + gamma((yA(i) - yB(i - 1)) / b)

Next i

'The final atom in A has an in-plane strain term and a misalignment term

Ui = Ui + (yA(noatoms) - yA(noatoms - 1) - b) ^ 2

Ux = Ux + gamma((yA(noatoms) - yB(noatoms - 1)) / b)

End Sub

Private Sub Calcw()

' This subroutine is unchanged since the earliest WJC versions

' except that it calls the subroutine CalcEnergy to calculate

' the energy at each stage

wmin = 0.01

wmax = 8

w1 = (wmin + wmax) / 2

w2 = wmax

Do While (w2 - w1) * (w2 - w1) > 0.000000000001

w = w1

Call CalcEnergy

U = Ui * A2 + Ux * A1

w2 = w1 * 1.01

w = w2

Call CalcEnergy

U2 = Ui * A2 + Ux * A1

If U2 < U Then

wmin = w1

Else

wmax = w1

End If

w2 = w1

w1 = (wmax + wmin) / 2

Loop

' Finally, set the width to the calculated value

' and call CalcEnergy one last time to return the minimum energy

w = w1

Call CalcEnergy

End Sub

Private Function gamma(phi)

' Returns misalignment energy in J m-2

' uncomment the appropriate gamma function for a fitted gamma surface or the Frenkel approximation

'gamma = C1 * (1 - Cos(2 * Pi * phi)) + C2 * (1 - Cos(4 * Pi * phi)) + C3 * (1 - Cos(6 * Pi * phi))

'gamma = C1 * (1 - Cos(Pi * phi)) + C2 * (1 - Cos(2 * Pi * phi)) + C3 * (1 - Cos(3 * Pi * phi)) + C4 * (1 - Cos(4 * Pi * phi)) + C5 * (1 - Cos(5 * Pi * phi)) + C6 * (1 - Cos(6 * Pi * phi))

gamma = G * b / (40 * Pi ^ 2 * d_b) * (1 - Cos(2 * Pi * phi)) ' Frenkel

End Function

Sub Plot_phi()

' For testing misalignment function

' Calculates misalignment energy and its first 3 components for phi/b from 0 to 1

Call LoadInput

For i = 0 To 100

Sheet3.Cells(i + 2, 1) = i / 100

Sheet3.Cells(i + 2, 2) = gamma(i / 100)

Sheet3.Cells(i + 2, 3) = C1 * (1 - Cos(2 * Pi * i / 100))

Sheet3.Cells(i + 2, 4) = C2 * (1 - Cos(4 * Pi * i / 100))

Sheet3.Cells(i + 2, 5) = C3 * (1 - Cos(6 * Pi * i / 100))

Next i

End Sub

Sub Batch()

For m = 1 To Sheet4.Cells(1, 3)

Sheet1.Cells(2, 2) = Sheet4.Cells(m + 3, 3) 'a

Sheet1.Cells(3, 2) = Sheet4.Cells(m + 3, 4) 'b

Sheet1.Cells(4, 2) = Sheet4.Cells(m + 3, 5) 'd/b

Sheet1.Cells(5, 2) = Sheet4.Cells(m + 3, 6) 'E

Sheet1.Cells(6, 2) = Sheet4.Cells(m + 3, 7) 'nu

Sheet1.Cells(7, 2) = Sheet4.Cells(m + 3, 8) 'G

Sheet1.Cells(12, 2) = Sheet4.Cells(m + 3, 9) 'C1

Sheet1.Cells(13, 2) = Sheet4.Cells(m + 3, 10) 'C2

Sheet1.Cells(14, 2) = Sheet4.Cells(m + 3, 11) 'C3

'Sheet1.Cells(15, 2) = Sheet4.Cells(m + 2, 30) 'C4

'Sheet1.Cells(16, 2) = Sheet4.Cells(m + 2, 31) 'C5

'Sheet1.Cells(17, 2) = Sheet4.Cells(m + 2, 32) 'C6

Call Calculate

Sheet4.Cells(m + 3, 12) = Sheet1.Cells(12, 6) 'Up/Gb^2

Sheet4.Cells(m + 3, 13) = Sheet1.Cells(14, 6) 'tp/G

Sheet4.Cells(m + 3, 14) = Sheet1.Cells(16, 6) 'w0/b

Sheet4.Cells(m + 3, 15) = U 'Write out the energy of the atomic configuration

Sheet4.Cells(m + 3, 16) = Sheet1.Cells(15, 6) 'tp/MPa

Next m

End Sub

Sub PlotAtoms()

Call LoadInput

ReDim xA(noatoms)

ReDim xB(noatoms - 1)

ReDim dA(noatoms)

ReDim dB(noatoms - 1)

ReDim yA(noatoms)

ReDim yB(noatoms - 1)

a = Sheet5.Cells(1, 6)

Call Calcw

For i = 0 To noatoms - 1

Sheet5.Cells(i + 2, 1) = yA(i)

Sheet5.Cells(i + 2, 2) = yB(i)

Next i

Sheet5.Cells(noatoms + 2, 1) = yA(noatoms)

End Sub

## 6. Input Data for the DFT Calculations for Ti3SiC2

# -----------------------------------------------------------------------------

# Ti3SiC2 MAX phase

# P.R. Howie, R.P. Thompson, W.J. Clegg

# -----------------------------------------------------------------------------

# This is an input file for the density functional theory

# package SIESTA for Ti3SiC2. For more detailed information see :

# http://departments.icmab.es/leem/siesta/

# The file includes the species present, an optimised basis set for

# psuedopotentials generated according to the SIESTA manual, the details of the unit cell, parameters for the DFT simulation like sampling density and the types of output to record. Strains are applied by editing the unit cell and the atomic paramters as appropriate.

#

#

# First the system is named and described

SystemName Ti3SiC2

SystemLabel Ti3SiC2

NumberOfAtoms 12

NumberOfSpecies 3

LongOutput

# Here the species present are defined, along with atomic numbers and labels

%block ChemicalSpeciesLabel

1 22 Ti

2 14 Si

3 6 C

%endblock ChemicalSpeciesLabel

# The basis set describes the cut off for the psuedopotentials that are used to avoid the need to simulate all the core electrons. The psuedopotentials and the basis set were generated and optimised with to the method set out in the SIESTA manual.

%block PAO.Basis

Ti 4

n=4 0 2 E 33. 4.6

5.6980794 3.6439493

n=3 1 1 E 15. 4.6

4.5173556

n=4 1 1 E 60. 0.6

5.5386496

n=3 2 2 E 80. 5.5

6.4553993 3.7954099

Si 3

n=3 0 2 E 33. 4.6

7.1011920 4.8958556

n=4 0 1 E 33. 4.6

5.6676308

n=3 1 2 E 60. 0.6

7.1051600 4.4845347

C 3

n=2 0 2 E 33. 4.6

6.0636809 4.5192225

n=3 0 1 E 33. 4.6

4.6955906

n=2 1 2 E 60. 0.6

7.1011920 3.9995051

%endblock PAO.Basis

# The unit cell is defined by one length and three lattice vectors defined in Cartesian space.

LatticeConstant 3.0821 Ang

%block LatticeVectors

1.0 0.0 0.0

-0.5 0.866025 0.0

0.0 0.0 5.7462

%endblock LatticeVectors

# These parameters relate to the DFT simulation and include sampling density, the functionals used for exchange interactions etc. See the SIESTA manual for further details.

MeshCutoff 400.0 Ry

kgridcutoff 15. Ang

xc.functional GGA

xc.authors PBE

MaxSCFIterations 150

DM.MixingWeight 0.03

DM.NumberPulay 3

SolutionMethod diagon

ElectronicTemperature 25 meV

# Here the desired outputs that are defined, see the SIESTA Manual for details.

WriteMullikenPop 1

WriteHirshfeldPop .true.

WriteVoronoiPop .true.

SaveRho .true.

SaveDeltaRho .true.

SaveBaderCharge .true.

SaveInitialChargeDensity .true.

# THe Zmatrix defines the atomic positions and any contstraints that apply. The lines in "fractional" define the species, the x, y, & z coordinates and flags that either allow or disallow relaxation of those atomic coordinates. The "variables" and "constraints" are used to apply any constraints that apply by symmetry of the unit cell.

%block Zmatrix

fractional

1 0. 0. 0. 0 0 0

3 0.666667 0.333333 Z1 0 0 0

1 0.333333 0.666667 Z2 0 0 0

2 0. 0. 0.25 0 0 0

1 0.333333 0.666667 Z3 0 0 0

3 0.666667 0.333333 Z4 0 0 0

1 0. 0. 0.5 0 0 0

3 0.333333 0.666667 Z5 0 0 0

1 0.666667 0.333333 Z6 0 0 0

2 0. 0. 0.75 0 0 0

1 0.666667 0.333333 Z7 0 0 0

3 0.333333 0.666667 Z8 0 0 0

variables

Z1 0.07272

Z2 0.1355

constraint

Z3 Z2 -1. 0.5

Z4 Z1 -1. 0.5

Z5 Z1 1. 0.5

Z6 Z2 1. 0.5

Z7 Z2 -1. 1.

Z8 Z1 -1. 1.

%endblock Zmatrix
